# Integrated Parenting Intervention for Depressed Fathers of Young Children: A Nested Qualitative Study From Pakistan

**DOI:** 10.1192/bjo.2024.166

**Published:** 2024-08-01

**Authors:** Rabia Sattar, Nasim Chaudhry, Tayyeba Kiran, Nusrat Husain, Ishrat Husain

**Affiliations:** 1 Pakistan Institute of Living and Learning, Lahore, Pakistan; 2 Pakistan Institute of Living and Learning, Karachi, Pakistan; 3 Pakistan Institute of Living and Learning, Rawalpindi, Pakistan; 4 University of Manchester, Manchester, United Kingdom; 5 Mersey Care NHS Foundation Trust, Liverpool, United Kingdom; 6 Campbell Family Mental Health Research Institute, Toronto, Canada; 7 University of Toronto, Toronto, Canada

## Abstract

**Aims:**

Depression is a leading cause of disability, contributing to the global burden of disease. Low- and middle-income countries (LMICs) carry over 80% of this disease burden. There are high rates of depression in men in Pakistan. Paternal depression is often overlooked and is an under-researched area. Fathers are at risk of depression particularly if their partner is depressed. There is a need for integrated partner inclusive interventions to improve both parent and child outcomes including overall child development. Therefore, this nested qualitative study aims to identify barriers in transition to scale up an innovative low-cost partner inclusive culturally adapted psychosocial intervention for depressed fathers through a process evaluation from the perspective of fathers.

**Methods:**

This qualitative study was nested within a cluster randomized controlled trial, with depressed fathers of young children (0–3 years) recruited from 18 towns in Karachi, Pakistan. Face to face or digitally audio-recorded interviews were conducted with depressed fathers (N = 24) from the intervention arm of the trial at end of intervention period (i.e., 4-month post-baseline). The intervention involved twelve sessions of a parenting intervention called Learning Through Play integrated with group Cognitive Behaviour Therapy and manual content from “Focus on Fathers”. Interviews were guided by a semi-structured topic guide to explore perceived usefulness of the intervention with particular focus on exploring any additional benefits or challenges of engaging partners into the intervention. Interviews lasted approximately an hour. Data were analysed using the principles of Framework Analysis. A concurrent analysis of initial interviews directed towards further interviews until data saturation.

**Results:**

Analysis highlighted perceived usefulness of the intervention such as improvement in mood, engagement in routine tasks, healthy thinking patterns, increased attachment with child, improved relationship with the family, improvement in parenting knowledge and more positive attitudes towards child development, supporting partners in household chores, and recommendation to promote this partner inclusive parenting program throughout the country.

**Conclusion:**

Addressing depression in parents is hugely important due to its adverse impact on both parents and children. This low-cost parenting program supported fathers in their parenting role along with improvement in psychological well-being. This has also informed barriers and facilitators to implement the LTP plus parenting program and the possibilities to roll out the intervention at national level.

